# Comprehensive identification of the *PEBP* gene family in *Vicia faba* highlighting *VfTFL1a* variation and phenotypic insights into determinate growth

**DOI:** 10.1038/s41598-025-12864-0

**Published:** 2025-08-06

**Authors:** Hannah Ohm, Umer Mahmood, Jenny Östberg, Josefin Alverup, Åsa Grimberg, Per Hofvander

**Affiliations:** Department of Plant Breeding, Swedish University of Agricultural Sciences (SLU), SE-234 22 Lomma, Uppsala, Sweden

**Keywords:** *PEBP*, Determinate growth, Terminal inflorescence, TFL1, *Vicia faba*, Biotechnology, Genetics, Plant sciences

## Abstract

The phosphatidylethanolamine binding protein (PEBP) family regulates key plant processes including growth, development, flowering, seed germination, formation, and dormancy. Despite their importance for these processes that determine agronomical important characters, *PEBP* genes in faba bean (*Vicia faba* L.*)* and closely related legumes remain underexplored. This study identified 11 *VfPEBP* genes for the first time in *Vicia faba*, classifying them into *MFT*-like, *TFL*-like, and *FT*-like subfamilies, and examining their relationships to *PEBP*-genes of the legumes *Pisum sativum* and *Vicia sativa. TFL1* homologs, crucial for growth determinacy, are an important focus for breeding to achieve a more confined flowering and maturation time. Cis-element analysis suggested *VfTFL1*-genes are regulated by light, hormones, and abiotic stress. Amplicon sequencing of *VfTFL1a*, a gene linked to shoot apical meristem fate, identified novel allelic variation but none of these could discriminate determinate from indeterminate varieties, invalidating a previously reported marker. Field trials revealed that determinate varieties of faba bean flowered later but had more uniform agronomic traits compared to indeterminate ones. These findings provide new insights into the *PEBP* gene family, highlighting its critical role in regulating flowering time and plant architecture in *Vicia faba* and underscoring its potential as a target for crop improvement.

**Supplementary Information:**

The online version contains supplementary material available at 10.1038/s41598-025-12864-0.

## Introduction

Faba bean (*Vicia faba *L.), a grain legume of significant agricultural importance, is renowned for its high seed protein content and adaptability to various environmental conditions, making it a valuable crop for sustainable agriculture and a promising food source for a healthy diet^1^. The plant architecture of faba bean, affecting traits such as determinacy, branching, lodging, flowering time, pod setting, and maturation time, plays a crucial role in its overall productivity. Understanding the genetic and molecular basis of plant architecture in faba bean can therefore lead to significant advancements in breeding and agricultural practices. Central to plant development and architecture are the phosphatidylethanolamine-binding protein (*PEBP*) genes. Particularly the balance between *FT*, which promotes flowering, and *TFL1*, which represses it, modulates plant architecture by determining whether the meristem adopts indeterminate or determinate growth^2^. Although most faba bean varieties are indeterminate, some varieties exhibit determinate growth, characterized by the formation of a terminal inflorescence at the shoot apical meristem (SAM) that halts further plant growth, leading to a limited flowering time and more condensed seed maturation.

 In plants, the *PEBP* gene family stands out due to its highly conserved nature and multifaceted functions; the genes in this family are involved in flower induction, determination of plant architecture, embryogenesis, stress tolerance, hormone signaling, and tuberization^3^^4^. The PEBP family can be divided into the three subfamilies MOTHER OF FT AND TFL1 (MFT)-like proteins, TERMINAL FLOWER1 (TFL1)-like proteins, and FLOWERING LOCUS T (FT)-like proteins^5^. Many plant species, especially crop plants, have experienced an expansion of the *PEBP* gene family through whole-genome and segmental duplications, leading to functional diversification. Extensive research on individual *PEBP* genes across various plant species has revealed that dicotyledons typically possess six to nine *PEBP* genes, while monocotyledons exhibit approximately three times this number^6^.

In legumes, the *FT* and the *TFL1*-like genes are conserved but exhibit a substantial expansion in number compared to Arabidopsis, with some members displaying notably divergent sequences^7^^8^. For example, there are five orthologs of *TFL1* identified in chickpea (*Cicer arietinum*)^9^ and four in soybean (*Glycine max*)^10^. Three orthologs of *TFL1* have been described in pea (*Pisum sativum*), one of which was found identical to the already known gene *DETERMINATE* (*TFL1*a/*DET*) and another one corresponding to the late flowering gene (*TFL1c/LF*). While the function of *PsTFL1b* remains uncharacterized^11,12^, the corresponding *GmTFL1b* has been identified as the key gene responsible for determinate plant architecture in soybean^13^. Three distinct subclades of *FT* (*FTa*,* FTb*, and *FTc*) genes are found in legumes^8^. In Arabidopsis, FT, and TFL1 are relatively short proteins (175 and 177 amino acids long, respectively) showing a high degree of sequence similarity (only 39 non-conserved amino acids) despite their opposite functions in flowering. In fact, a single amino acid substitution can reverse their respective functions^14^. Loss-of-function mutants of *TFL1* in Arabidopsis exhibit early flowering, with a terminal inflorescence^15^. Conversely, over-expression of *TFL1* significantly prolongs all developmental phases, showing that *TFL1* functions to maintain the SAM in an indeterminate state by delaying the activation of genes responsible for floral meristem identity^16^. Just as for Arabidopsis, a mutation in the orthologous *TFL1* gene of several legumes has been shown to increase the expression of *LEAFY* (*LFY*) and *APETALA* (*AP1*), resulting in a determinate growth of the inflorescence and the development of a terminal floral meristem preventing further SAM maintenance^7,12,17^.

In this study, members of the *PEBP* gene family in *Vicia faba* were identified and systematically analyzed at the genome-wide level for the first time, including their phylogenetic relationships, gene structure, and comparison to other Fabaceae species. Genetic variations in *VfTFL1a* were examined in a panel of eight *Vicia faba* varieties, encompassing determinate types, characterized by terminal inflorescences, and indeterminate types, which exhibit continuous leaf development at the shoot apical meristem. Additionally, field data on phenotypic variations in determinate and indeterminate varieties were assessed. *PEBP* genes were also identified in *Vicia sativa*, the closest relative of *Vicia faba* with a sequenced genome^18^. This study aimed to provide deeper insights into the evolutionary dynamics of the PEBP family within leguminous species.

## Material & Methods

### Plant material and DNA extraction

For genotypic comparisons, four indeterminate varieties Aurora, Mikko, Fanfare, and Taifun, and four determinate varieties Ticol, Tinova, Tina, and Bruno were used. All seeds were kindly provided from the John Innes Centre (Norwich, England), NordGen (Alnarp, Sweden), and Lantmännen (Svalöv, Sweden).

Seeds were sown in plastic pots with nutrient soil (50% peat, pH 5.5–6.5, added per m^3^ soil: 5.5 kg lime, NPK 11-5-18 kg, 200 g micronutrients and 100 g iron) under greenhouse conditions: 18–21 °C light 6–22 h < 200 W/m^2^. Young leaves were cut from plants and stored at −80 °C, until ground to a fine powder in liquid nitrogen in steel containers with 2 mm Ø steel beads, using a mixer mill (MM 400, Retsch GmbH, Haan, Germany) at 30 Hz. Genomic DNA from frozen leaf tissue was extracted using the Plant Mini Kit (Qiagen, Valencia, CA, USA) according to the manufacturer’s protocol. Complementing plants were germinated on media, and their DNA was extracted using the Nucleospin^®^ Plant II kit (MACHERY-NAGEL), following previously established protocols^19^.

### Sequence isolation and characterization

*Vicia faba* DNA sequences corresponding to *AtTFL1* were amplified by PCR (Unocycler, VWR) using specific primers designed based on the published reference genome Vf var. Hedin/2 and var. Tiffany or primers derived from Avila et al. 2006 and 2007. For primer sequence and PCR settings, see Supplementary Table S1. The amplified products were cloned into pJET1.2-vector (Thermo Scientific, Massachusetts, USA) and sequenced using Sanger sequencing (Eurofins, Ebersberg, Germany). Sequence characterization, multiple sequence alignments, and visualizations were made using the software CLC Main Workbench 21.0.3 (Qiagen, Valencia, CA, USA).

### Identification of PEBP gene family members in Vicia faba

This study used genetic data from four plant species—three legumes (*Vicia faba*, *Vicia sativa*, and *Pisum sativum*) and the model species *Arabidopsis thaliana*—to perform a comparative evolutionary analysis of the *PEBP* gene family. Protein and coding sequences were retrieved from publicly available reference genomes of *Arabidopsis thaliana* (TAIR11), *Pisum sativum* (GCA_024323335.2), *Vicia faba* var. Hedin/2 (GCA_948472305.1), and *Vicia sativa* (GCA_021764765.1). The *A. thaliana* genome was obtained from The Arabidopsis Information Resource (TAIR, http://www.arabidopsis.org), *P. sativum* and *V. faba* from NCBI, and *V. sativa* from the GigaDB database (http://gigadb.org/dataset)^18,20,21^.

PEBP family proteins containing the conserved PEBP motif (Pfam ID: PF01161.24) and the Hidden Markov Model (HMM) profiles were acquired from the HMMER web server (https://www.ebi.ac.uk/Tools/hmmer/). All *PEBP* genes were mined using two steps. First, the HMM profile was used as a query to search the protein databases of all species using HMMER V3.0 to identify candidate genes with the default “inclusion threshold”^22^. The Basic Local Alignment Search Tool Protein (BLASTP) program was used to search against the *Arabidopsis thaliana* proteome database using retained candidate genes to confirm their identity as *PEBP* genes, with an E-value of 1e^−5^ and a minimum alignment coverage of 50%^23^. The nomenclature for *PEBP* genes in other species was based on the corresponding *AtPEBP* orthologues.

### Phylogenetic analysis

To explore the phylogenetic relationships of PEBP family members, a maximum likelihood (ML) tree was generated using IQ-TREE v2.3.5 based on the deduced protein sequences of these genes in examined species, with the Q.plant + G4 model^24^. To validate the ML tree, a neighbor-joining (NJ) tree was constructed using MEGA 7.0 (Molecular Evolutionary Genetics Analysis, Tokyo Metropolitan University, Japan) based on PEBP proteins from the plant species examined. Bootstrap analysis with p-distance and pairwise deletion was conducted with 1,000 replicates^25^. Multiple sequence alignments of PEBP proteins were performed using MUSCLE with default parameters^26^. The resulting phylogenetic tree was visualized using FigTree v1.4.4 (http://tree.bio.ed.ac.uk/software/figtree/).

### Protein conservative domain and gene structure analysis

The Multiple Expectation Maximization for Motif Elicitation program (MEME v5.5.7, http://alternate.meme-suite.org) suite was used to analyze the conserved motifs of PEBPs with the following settings: minimum and maximum motif width 6 and 100, respectively, and 10 number of motifs. The motifs in the protein database were applied using MAST 4.12.0^27^. Exon-intron organization of *PEBP* was analyzed using Gene Display Server 2 (http://gsds.cbi.pku.edu.cn/)^28^.

### Analysis of TFL1

A 1500 bp genomic sequence located both upstream and downstream of the transcribed region of the *TFL1a* and *TFL1c* genes was analyzed to identify potential cis-elements. Cis-acting elements were obtained using the PlantCARE online software (https://bioinformatics.psb.ugent.be/webtools/plantcare/html/) and supplemented with manually added elements known to be important in the *PEBP* family based on literature^29^. The data was visualized with TBtools v2.119. The alignment of the non-coding sequences from the different *TFL1* orthologues was carried out using mVISTA (http://genome.lbl.gov/vista/index.shtml)^30^.

### Chromosomal location analysis

The chromosomal distribution map was generated and visualized using MapChart v.2.32^31^, with sequence position data obtained from the Ensembl Plants database (release 45)^32^ and chromosome information sourced from the European Nucleotide Archive (ENA) (https://www.ebi.ac.uk/ena/browser/home).

### Field trial and phenotyping data

Field experiments were conducted at the SITES Research Station Lönnstorp, Swedish University of Agricultural Sciences (SLU), in Alnarp (55.65° N, 13.06° E) over two consecutive growing seasons, 2021 and 2022. The field trials were used for a characterization of a diversity panel of faba bean with 220 different varieties^33^, among which the varieties used in this study were included and phenotyped in detail with regard to plant architecture traits. Each plot, measuring 1 m x 0.75 m, was hand-sown with fifty seeds of a single variety, with each variety represented in duplicate plots. For details on field experimental design, management, and weather conditions see Ohm et al. (2024). Growth behavior was assessed through visual inspection of plants towards the end of the flowering period, categorizing them as either determinate (with a terminal inflorescence on the main stem) or indeterminate (with continuous leaf development at the top of the main stem). Plant height was measured as the average height of five randomly selected plants per plot ~ 75 days after sowing. The number of days from sowing to flowering was recorded when 50% or more of the established plants in a plot had at least one open flower. Similarly, the number of days from sowing to maturity was noted when 50% or more of the established plants in a plot had filled, dry, and brown/black pods.

Seed size and thousand-grain weight (TGW) were determined using 100–200 seeds with the seed analyzer MARViN ProLine I (Marvitech, Germany). The yield parameter, expressed as grams of seeds per plant (YIELD), was calculated by dividing the total weight of harvested seeds from a plot by the number of established plants in that plot. The number of seeds per plant (SEEDS) was determined by dividing the total weight of harvested seeds from a plot by its TGW, multiplying by 1000, and then dividing by the number of established plants in that plot.

### Statistical analysis of field data

Descriptive statistics were used to compare the phenotypic traits between the indeterminate and determinate varieties from the field study. Statistical analysis was conducted on the average values for each variety, calculated across two replicates and two seasons. Student’s t-test determined the statistical significance of differences; the alpha level was set to *p* < 0.05. Data visualization was conducted using the ggplot2 package in R^34^.

## Results

### In silico identification and characterization of PEBP family genes in Vicia faba

Through an HMMER search targeting the conserved PEBP domain and subsequent BLASTP alignment, we initially identified 47 gene members across four plant species. However, three sequences were excluded from further analysis due to having over 50% gaps or ambiguous regions in the evolutionary analysis. The remaining sequences comprised six members in *Arabidopsis thaliana*, 15 in *Pisum sativum*, and 11 members each in *Vicia sativa* and *Vicia faba* (Supplementary Table S2. The protein lengths of PEBP family members ranged from 173 to 177 amino acids in *A. thaliana*, 162–188 in *Pisum sativum*, 134–180 in *Vicia faba*, and 130–224 in *Vicia sativa*. The 11 *VfPEBP* genes were named according to their similarity with the orthologous *Arabidopsis thaliana* or *Pisum sativum* genes^35^, defining six *VfFT*, three *VfTFL1*, one, and one *VfMFT*. The expansion and structure of *PEBP* family members in several legume species as compared to the model plant Arabidopsis is highlighted in Table 1. The *PEBP* genes identified in this study are marked in bold; all other genes were obtained from existing literature^35–40^.

**Table 1:** Classification of *PEBP* genes in *Arabidopsis thaliana* and six legume species. Gene names in bold indicate that they are part of the results in this study, while others are retrieved from literature for clarity and consistency, a standardized nomenclature was adopted in this study, despite variations found in the literature^35–40^. Notably, *FT* was previously referred to as *FTLe* in Medicago and *FTL* in Pisum. The accession number or protein sequence for each gene can be found in supplementary Table S2.

	*Arabidopsis thaliana*	*Vigna radiata*	*Medicago truncatula*	*Vicia faba*	*Vicia sativa*	*Cajanus cajan*	*Pisum sativum*	*Glycine max*
MFT-like	*MFT*	*VrMFT1*	*MtMFT*	**VfMFT**	**VsMFT**	*CcMFT*	*PsMFT*	*GmMFTa* *GmMFTb*
	*BFT*		*MtBFT*	**VfBFT**	**VsBFT**		*PsBFT*	*GmBFTa* *GmBFTb*
*TFL1*-like	*ATC/CEN*							
	*TFL1*	*VrTFL1* *VrTFL2* *VrTFL3*	*MtTFL1a* *MtTFL1b* *MTTFL1c*	**VfTFL1a** **VfTFL1b** **VfTFL1c**	**VsTFL1a** **VsTFL1b** **VsTFL1c**	*CcTFL1-1* *CcTFL1-2* *CcTFL1-3* *CcTFL1-4*	*PsTFL1a/DET* *PsTFL1b* *PsTFL1c/LF*	*GmTFL1a* *GmTFL1b* *GmTFL1c* *GmTFL1d*
*FT*- like	*FT* *TSF*	*VrFT1* *VrFT-like1* *VrFT-like2*	*MtFTa1* *MtFTa2* *MtFTa3* *MtFTb1* *MtFTb2* *MtFTc*	**VfFTa1** **VfFTa3** **VfFTb1-1** **VfFTb1-2** **VfFTb2** **VfFTc**	**VsFTa1** **VsFTa2** **VsFTa3** **VsFTb1** **VsFTb2** **VsFTc**	*CcFT1* *CcFT2* *CcFT3* *CcFT4* *CcFT5* *CcFT6* *CcFT7* *CcFT8*	*PsFTa1/GIGAS* *PsFTa2* **PsFTa2-2** **PsFTa2-3** **PsFTa2-4** **PsFTa3** *PsFTb1* *PsFTb1-2* *PsFTb2* *PsFTc*	*GmFT1a* *GmFT1b* *GmFT2a* *GmFT2b* *GmFT2c* *GmFT3a* *GmFT3b* *GmFT5a* *GmFT5b* *GmFT4* *GmFT6* *LJ18*

**Fig. 1:**
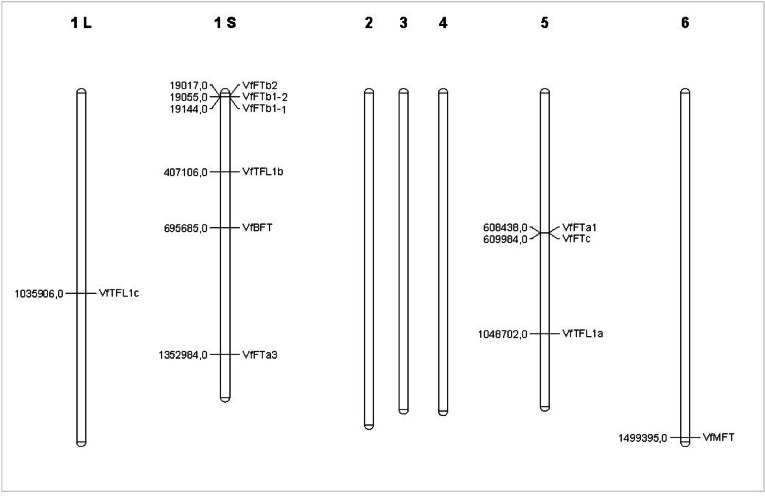
Chromosomal distribution and localization of *VfPEBP* genes in faba bean. Black vertical lines represent the six faba bean chromosomes, labeled at the top. The positions of the *VfPEBP* genes are marked along the chromosomes with horizontal lines. Chromosome positions are given in kbp.

To understand the distribution patterns of the *PEBP* gene family across the genome, genomic annotation data from faba bean was utilized to determine the chromosomal positioning of the *VfPEBP* genes. As shown in Fig. 1, the 11 genes are spread across the four chromosomes, 1 S, 5, 6 (and possibly 1 L). Chromosome 1 S holds the largest number, with six *VfPEBP* genes, while chromosome 5 contains three. In contrast, only one *VfPEBP* gene is located on chromosome 6, and possibly another on chromosome 1 L.

### Phylogenetic and structural analysis of PEBP family genes

**Fig. 2:**
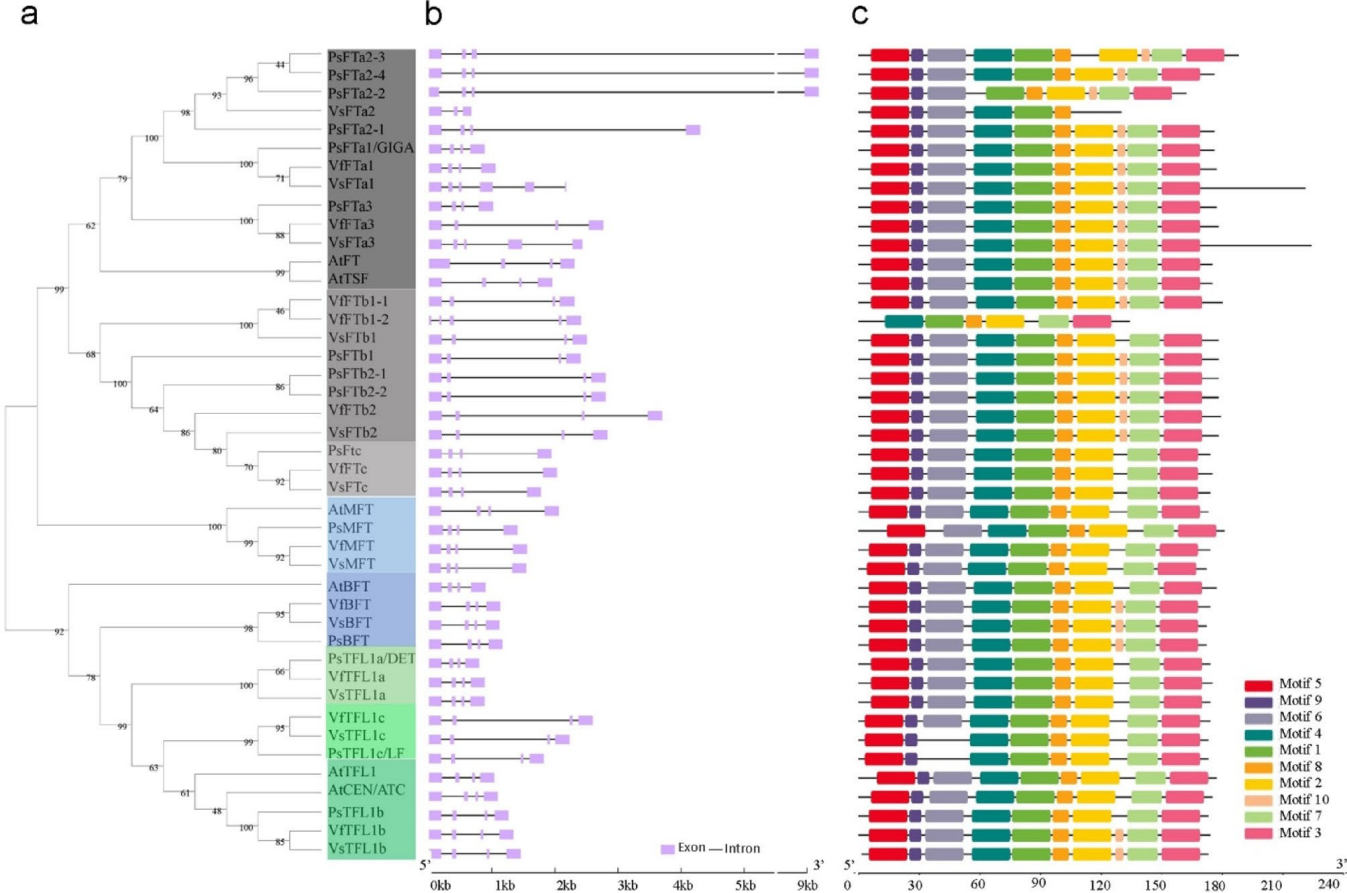
Phylogenetic and structural analysis of *PEBP* family genes. (**a**) Phylogenetic tree of PEBP family proteins from *Vicia faba* (Vf), *Arabidopsis thaliana* (At), *Vicia sativa* (Vs), and *Pisum sativum* (Ps), illustrating their evolutionary relationships. (**b**) The corresponding intron-exon structures of each DNA sequence are shown alongside the tree. (**c**) Conserved amino acid motifs among the PEBP sequences are depicted and color-coded (Motif 1–10), highlighting shared functional regions across species. Motif sequences are found in Supplementary Table S3.

To further investigate the relationship among *PEBP* family members in *Vicia faba* and other species, a phylogenetic tree was constructed using the full-length PEBP protein sequences from *Vicia faba*,* Pisum sativum*,* Arabidopsis thaliana*, and *Vicia sativa* (Fig. 2a). The analysis identified the three distinct subgroups of genes: *FT*-like, *TFL1*-like, and *MFT*-like. In *Vicia faba*, six genes were classified as *FT*-like, which is a higher number compared to *Arabidopsis thaliana* (three) and equal to *Vicia sativa* (six), but fewer than *Pisum sativum* (ten). Three genes in *Vicia faba* were categorized as *TFL1*-like, a consistent number among legumes investigated, with the addition of *TFL1b* compared to Arabidopsis. One gene in *Vicia faba* was categorized for each *BFT* and *MFT*, consistent with the numbers found in most other species, except for *Pisum sativum*, which possesses two *BFT* genes.

To explore the structural diversity of *VfPEBP* genes, the number of exons and introns, and the distribution of conserved domains were investigated (Fig. 2b and c). The gene lengths, including introns, generally ranged from 1 kb to 4 kb, with the notable exception of the three *PsFT2* genes, which extend beyond 9 kb. Most *PEBP* genes are composed of four exons and three introns, with substantial variation observed in the lengths of introns 2 and 3. Eight conserved amino acid motifs could be found in all PEBPs and occurring in the same order across the proteins, with a single exception (*VfFTb1-2*). Additionally, ten motifs were identified in the majority of the proteins (Supplementary Table S3). All *Vicia faba* PEBP proteins contain one or both of the highly conserved motifs, DPDxP and GxHR (part of motif 4 and 2, respectively, in Fig. 2c), which likely contribute to the ligand binding pocket conformation^41^ (Supplementary Figure S4). The conserved motif DPDxP is present in almost all sequences, except for the FTb-proteins in each of *Pisum sativum*, *Vicia faba*, and *Vicia sativa* that instead possess an alternative domain, NPDxP. Additionally, the FTc sequence exhibited a variation in the motif, changing to DADxP. The conserved motif GxHR is present across all sequences except for VsFTa2. An amino acid residue at alignment position 96, which alternates between tyrosine (Y) in FTs and histidine (H) in TFL1 proteins, are observed in our data in agreement with previous reports in other plants. This single amino acid serves as a distinguishing feature separating FT from TFL1, converting the flowering activator to a repressor^14^ (Supplementary Fig. S4). Interestingly, this distinction also applies to BFT, except for in *Arabidopsis thaliana*.

### Analysis of putative cis-acting regulatory elements

**Fig. 3:**
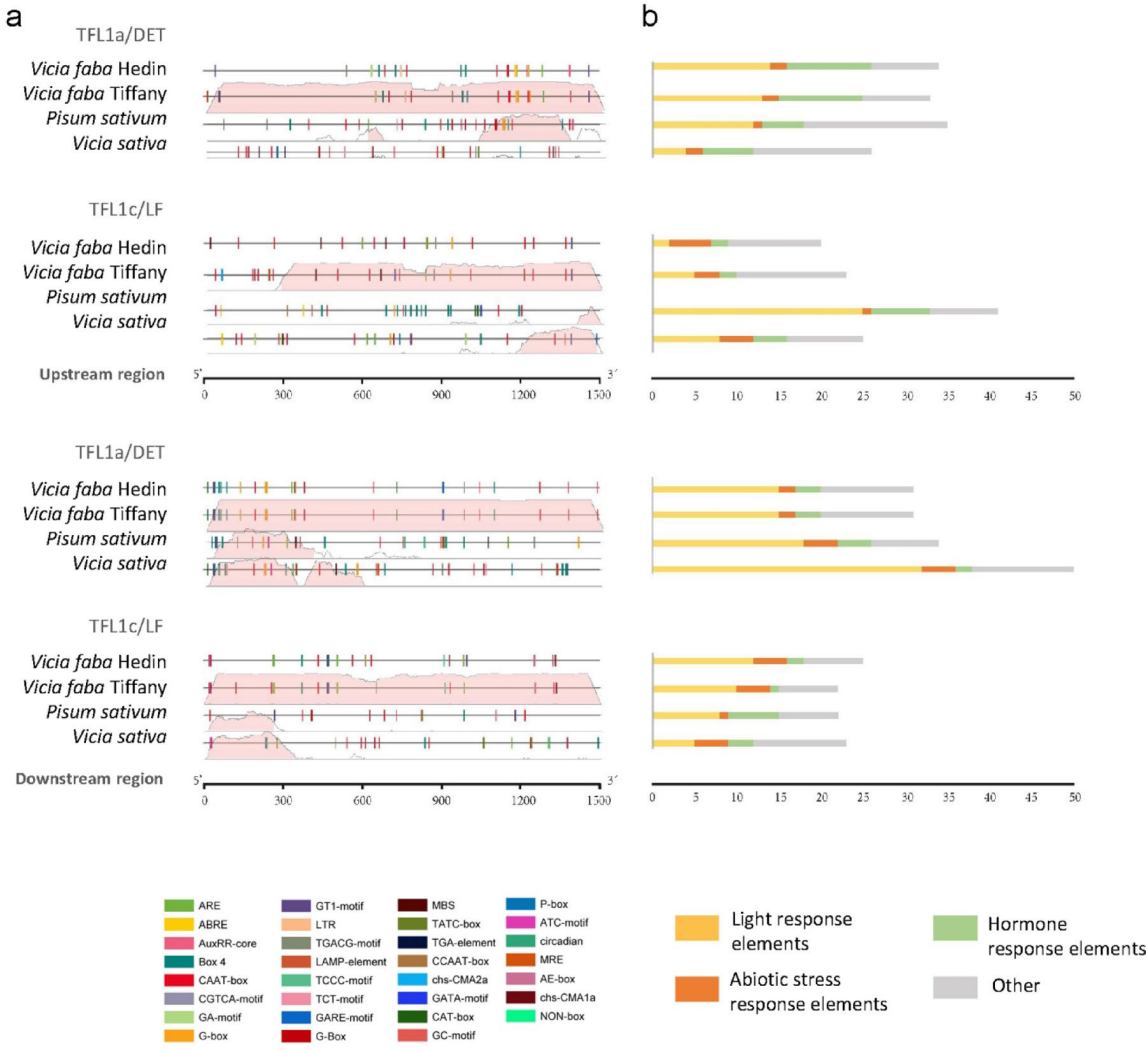
Predicted regulatory elements in *TFL1* genes. (**a**) Distribution of predicted regulatory elements in the 1500 bp upstream and downstream genomic regions of the *TFL1a* and *TFL1c* genes in *Vicia faba* (two reference varieties; Hedin and Tiffany), *Pisum sativum*, and *Vicia sativa* species. Color-coded captions show element names. Sequences were aligned to the sequence of *Vf* Hedin and the graphical output in the background shows base pair identity to *Vf* Hedin (0-100% range) in a sliding window of 1500 bp, > 75% similarity in red. (**b**) Bar graphs showing the count of elements associated with light response (yellow), hormone response (green), abiotic stress response (orange), and others (grey) for each *TFL1* gene. All cis-acting elements are further detailed in Table S5.

The *TFL1* genes *TFL1*a and *TFL1*c, also known as *DET* and *LF* in *Pisum sativum*, which play a critical role in inhibiting meristem inflorescence development and thus regulating plant architecture, were investigated in more detail based on reference genomes. Genomic regions of 1500 bp flanking the coding sequences, both upstream and downstream of their homologs in *Vicia faba*, *Pisum sativum*, and *Vicia sativa* were mapped for predicted cis-regulatory elements (Fig. 3a). These elements were further categorized by function (Fig.3b). Our analysis focused on predicted DNA transcription factor binding sites derived from conserved short motifs relevant to *TFL1*’s biological functions, including responses to light, hormones, stresses, and other signaling pathways. Out of 54 motifs searched, 30 were predicted to be present in the up-and downstream regions of the *TFL1* genes (detailed in Supplementary Table S5). Among these, Motif Box 4, part of a conserved module involved in light responsiveness, and the CAAT box, a common cis-acting element in promoter and enhancer regions, were the most prevalent. In *Vicia faba*, approximately 40% of the motifs in both the upstream and downstream regions are categorized as light-responsive, highlighting the potential significance of photo response regulation in *TFL1* gene expression across species. Several cis-acting elements were found to be of particular interest as they coincided between species and regional location (highlighted in Supplementary Figure S6), which suggests that these could be of relevance for the control of *TFL1*.

### Determinacy by DET mutations in faba bean

To investigate mutations that potentially affect the function of *TFL1a* and *c* genes that could explain a determinate phenotype (i.e. showing terminal inflorescence) in faba bean, we isolated, cloned, and sequenced the coding region, including 500 bp up-and downstream of the gene. We analyzed eight varieties, comprising four individuals from each phenotypic group (indeterminate and determinate). The isolation and sequencing of the *VfTFL1a* coding sequence, including upstream and downstream regions, revealed several sequence variations among the investigated varieties (Supplementary Figure S7). We observed 1–37 single nucleotide polymorphisms (SNP) and insertion/deletions throughout the sequence, with five variations in the transcribed region of the gene confined to the first exon and intron. Notably, none of these variations could be linked to distinguishing determinate varieties from indeterminate varieties. Further, we identified 35 nucleotide variations between the two reference genomes, Tiffany and Hedin (which both are indeterminate varieties), none of which were located within the transcribed region.

All primers utilized in this study are detailed in Supplementary Table 1. Due to the significant challenges encountered in isolating the target sequences from different *Vicia faba* varieties, we have also included the primers that were unsuccessful, to contribute to potential future refinements in methodology.

**Fig. 4:**
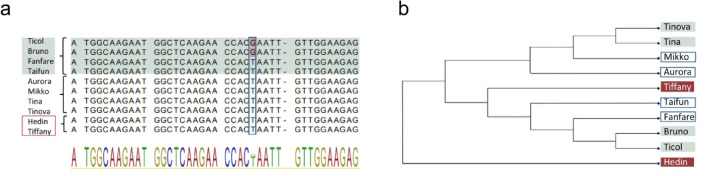
Allelic variation and phylogenetic relationships of the *VfTFL1a* gene in *Vicia faba* varieties with different phenotypes. (**a**) Multiple sequence alignment of the coding sequence for *VfTFL1a* from 10 *Vicia faba* varieties, including four determinate phenotype varieties (in light green) and six indeterminate phenotype varieties, including the reference varieties Hedin and Tiffany (in red). The SNP (G/T) at the 26th bp, previously identified as a diagnostic marker distinguishing the two phenotypes is highlighted. In this dataset, the SNP differentiates only half of the determinate varieties from the indeterminate varieties. For alignment of the entire sequence (transcribed region of the gene as well as 500 bp up-and downstream of it) see Supplementary Figure S7. (**b**) Phylogenetic relationships among the 10 varieties based on the transcribed region of the gene and 500 bp upstream and downstream of the *VfTFL1a* gene. The tree shows mixed branching patterns and does not group varieties strictly by phenotype, with determinate (light green) and indeterminate phenotypes (white, including reference varieties in red). The tree was constructed using the Maximum Likelihood method with Neighbor-Joining and the Kimura 80 substitution model in CLC^42,43^.

Previously, a SNP at bp position 26 in the coding sequence of the *VfTFL1* gene was reported by Avila et al. (2006 and 2007) to effectively distinguish determinate faba bean varieties from indeterminate ones^42,43^. However, our study, found that the previously reported SNP marker did not consistently correlate with the growth phenotype (Fig. 4a). Phylogenetic analysis of *VfTFL1a* sequences from the 10 varieties showed diverse allelic patterns with no clear clustering based on determinate versus indeterminate growth habit (Fig. 4b).Our observed genetic variations in *VfTFL1a* among varieties coincided with only one predicted regulatory motif, CCCAATTT (bp 149–156, upstream), which contains an A/C SNP at its first position. This variation occurs in both determinate and indeterminate varieties, suggesting possible regulatory relevance.

### Field phenotypic observations

**Fig. 5:**
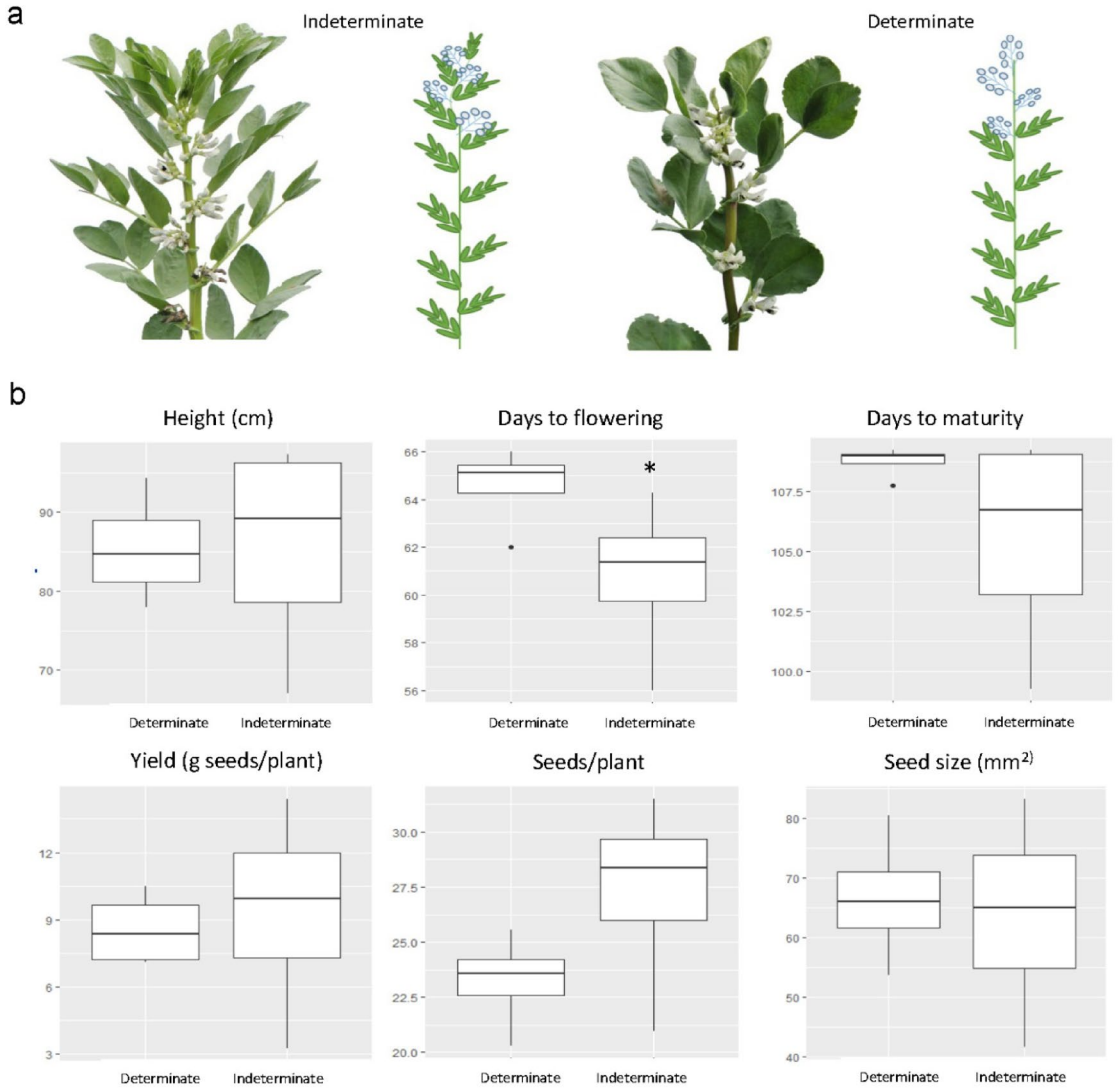
Growth habits and traits of indeterminate and determinate faba bean varieties. (**a**) Comparison of indeterminate (left) and determinate (right) faba bean varieties, photo and illustration. Images of all determinate varieties at the flowering stage are available in Supplementary Figure S9. (**b**) Boxplots illustrating the distribution of plant height, days to flowering, maturity, yield, seeds per plant, and seed size for both growth habits, based on data from a two-year field trial (Supplementary Table S8). Eight faba bean varieties were evaluated, with four representing each growth type. For each variety, 10–50 plants were assessed depending on the specific trait being measured. The values are the averages of each variety’s performance across the two years and both replicates, shown as mean (horizontal line), upper and lower quartiles (box), range (whiskers) and outliers (dots). Asterisk indicates significant differences according to Students t-test (*p* < 0.05).

To explore phenotypic differences between indeterminate and determinate faba bean varieties, we selected a group of indeterminate and determinate varieties, which were also used in the above-mentioned DNA sequence comparisons. The varieties were characterized for several agronomic traits in two years of field trials and their distinguishing plant architecture is shown in Fig. 5a and Supplementary Figure S9. Significant differences were observed in the number of days from sowing to flowering, with determinate varieties requiring a longer time to reach flowering. Even though not statistically significant, determinate varieties also showed a trend towards a longer time from sowing to maturity compared to indeterminate varieties. Determinate varieties exhibited a more condensed interval to maturity with less variability, in contrast to the greater variation observed for this trait in indeterminate varieties (Fig. 5b and Supplementary Table S8). While other traits did not show significant differences between determinate and indeterminate varieties, trends indicated that determinate varieties tended to have shorter plant height, lower seed yield, and fewer seeds per plant. Seed size, however, showed relatively similar characteristics between the two types. Overall, determinate varieties demonstrated reduced variability across all traits observed, compared to indeterminate varieties.

## Discussion

### Expansion and conservation of *PEBP* family genes in *Vicia faba*

The identification of 11 *PEBP* gene family members in *Vicia faba*—comprising six *VfFT*, three *VfTFL1*, one *VfBFT*, and one *VfMFT* gene—points to a significant expansion of this gene family in faba bean compared to *Arabidopsis*, particularly with respect to the *FT*-like genes. This expansion may underpin the more complex growth and flowering architecture of faba bean, allowing for functional specialization across gene copies. Unlike the simpler growth and flowering structure of Arabidopsis with its primary inflorescence, *Vicia faba* develops early lateral shoots, each with its inflorescences. The bushier growth form and more complex flowering pattern of faba bean suggest an evolutionary adaptation to optimize resource allocation, extending the flowering period and enhancing adaptability to diverse environmental conditions. The structural analysis of *VfPEBP* genes in this study revealed a high degree of conservation in terms of exon-intron organization, with most genes consisting of four exons and three introns. This structural consistency across species underscores the evolutionary importance of the *PEBP* family. However, the substantial variation observed in intron lengths, particularly in introns 2 and 3, may indicate potential regulatory differences among family members. The abundance of *FT*-like genes relative to *MFT*-like genes appears conserved across legumes, which aligns with findings by Pan et al., (2023) which suggests that species possessing numerous *FT*-like genes tend to exhibit fewer *MFT*-like genes^6^. This pattern may indicate an evolutionary relationship in which *MFT*-like genes could be ancestral to *FT*-like and *TFL*-like genes, with their divergence potentially coinciding with the origin of flowering plants. Interestingly, unlike in Arabidopsis, some of the numerous *FT* genes in soybean, for example, are expressed in the leaves while others are expressed in the shoot apical meristem (SAM) to regulate flowering. Su et al., (2024) suggest that genome duplication in soybean has altered the tissue-specific localization of the *FT* genes and their functional expression^37^. Future studies in faba bean could reveal a similar diversification within this and other subcategories of the *PEBP* gene family, with members functioning in various tissues of the plant. Alongside the ten conserved motifs identified in the PEBP individuals of *Vicia faba*, *Vicia sativa*, and *Pisum sativum*, the presence of specific motifs like DPDxP and GxHR in most VfPEBPs suggests functional conservation, as they are critical for ligand-binding pocket conformation. Variations in *FT*-like members both within different legume species and among the FT proteins themselves, such as the alternative NPDAP domain, may indicate functional specialization within this subfamily.

The uneven distribution of *VfPEBP* genes across four chromosomes in *Vicia faba*, with a notable concentration on chromosome 1 S, offers insights into the evolutionary dynamics of these genes. This pattern likely results from segmental duplication events, which are frequent in plant genomes and contribute to gene family expansion and functional diversification. The proximity of *VfFTb* genes mirrors the arrangement seen in *Medicago truncatula* and *Glycine max*, where *FTc* genes are located adjacent to *FTa* genes, suggesting a common origin from the same duplication event^35^.

In summary, the expansion and diversification of the *PEBP* gene family in *Vicia faba*, particularly the *FT*-like and *TFL1*-like genes, offer promising opportunities for targeted breeding strategies concerning flowering and plant architectural traits, which can be of high importance for agronomic performance. These findings also highlight the need for further research focused on the functional characterization of individual *VfPEBP* genes to elucidate their specific roles in controlling flowering time and plant architecture. Such studies will be essential for enhancing our understanding of the molecular mechanisms underlying these traits and for guiding future crop improvement efforts in *Vicia faba*. Additionally, it is important to address the inconsistent nomenclature of these genes, which varies not only among species, but even among studies on the same species, creating challenges in maintaining clarity and consistency. Future studies can help establish a standardized naming convention, facilitating clearer communication and more precise genetic information in this field.

### Phenotypic implications of determinate growth architecture in field

Plant architecture including the timing and distribution of reproductive structures are fundamental agronomic characteristics shaped by patterns of determinate and indeterminate growth. Mutations in *TFL1* could potentially lead to earliness, which is of great importance when cultivating the long-season crop faba bean in colder climate countries such as in the Nordic region. In our study, we selected eight varieties based on their growth habits: four determinate and four indeterminate. The varieties Mikko and Aurora were selected for their early maturity traits, essential for successful cultivation in the Nordic climate with its short growing season. Additionally, all varieties were chosen to ensure genetic diversity, as they are not closely related to each other. Field trials comparing determinate and indeterminate varieties revealed significant differences in earliness traits, with determinate varieties requiring a longer time from sowing until flowering. This aligns with the findings of Zhu et al. (2020), which show that TFL1 competes with FT to interact with transcription factor FD, thereby inhibiting the expression of meristem fate genes and delaying flowering^44^. While other traits such as plant height, maturity time, and seed yield only showed trends rather than significant differences between determinate and indeterminate varieties, the lower variability observed in determinate varieties across multiple traits suggests a more uniform growth pattern. This uniformity is particularly pronounced in the condensed maturation period (103–108 days for determinate varieties versus 96–108 days for indeterminate ones). Further, our observations aligned with previous studies indicating that determinacy often comes at the cost of yield reduction^44,45^.

### Molecular characterization of *TFL1* in *Vicia faba*

To elucidate the genetic basis of growth architecture in faba bean, we specifically examined the *TFL1*-like clade and identified three *TFL1* subclade members, designated as *VfTFL1a*, *VfTFL1b*, and *VfTFL1c*. The analysis of predicted cis-acting regulatory elements in the *TFL1* genes revealed a predominance of light-responsive elements, accounting for approximately 40% of the identified motifs. This finding highlights the probable importance of photoperiod in regulating *TFL1* expression and, consequently, plant architecture and flowering time in *Vicia faba*. The conservation of certain regulatory elements across species suggests their functional significance and potential targets for future breeding efforts, warranting further experimental validation.

Our sequence analysis of *VfTFL1a*, a homolog to the determinacy gene in other plant species, did not reveal distinguishing variations that could confirm the previously reported diagnostic SNP marker at position 26^42,43^. This discrepancy contrasts with the marker’s original validation across 36 European inbred lines, suggesting that its applicability may be limited to specific germplasm collections. The diverse allelic inheritance patterns observed among our varieties and the lack of clear phylogenetic clustering between determinate and indeterminate phenotypes indicate that the determinate trait likely arose independently multiple times through different genetic mechanisms. This suggests that the genetic control of determinacy in *Vicia faba* is more complex than previously thought, possibly involving multiple genetic factors, regulatory elements beyond the *VfTFL1a* coding sequence, or alternative molecular pathways across different breeding lineages.

### Evolutionary implications and future directions

The phylogenetic analysis of *VfTFL1a* sequences from different varieties did not show a clear separation between determinate and indeterminate phenotypes. This lack of clear clustering indicates that the determinate phenotype likely arose independently multiple times, possibly through different molecular mechanisms or regulatory changes. Genetic relatedness among the varieties could possibly have influenced the results for Avila et al. (2006, 2007), by limiting the genetic variability observed. Our varieties instead show a divergent background and a variation of the allelic heritage for *TFL1*^42,43^.

Given the distinct roles of *PsTFL1a/DET* on meristem fate and *PsTFL1c/LF* on flowering time in pea, it raises the question of whether *TFL1b* in pea as well as in faba bean might play a unique or complementary role in determination cues. Investigating *VfTFL1b* could provide valuable insights into the regulatory mechanisms governing SAM determinacy in legumes and shed light on evolutionary adaptations within the *TFL1* gene family. Previous studies in *Arabidopsis* have demonstrated that elements in the downstream sequence of *AtTFL1* influence meristem determinacy up to 3 kb^46^ from the coding region. Consequently, expanding the investigation to include further up- and downstream genomic regions of *Vf* genes may be relevant for identifying diagnostic markers that differentiate between determinate and indeterminate faba bean varieties. Warranting further investigation, this suggests that the key gene determining determinacy in faba bean may be one of the orthologs, *TFL1b* or *TFL1c*, or that the regulatory element responsible for the trait appears further up or downstream than 1 kb. Further, since it is known that the balance between the two homologous proteins, FT and TFL1, controls the SAM fate it is also important to look into *FT* and its potential functional mutations or affecting expression levels^14^.

### Challenges in gene isolation

The successful retrieval of specific gene sequences from the *Vicia faba* genome through PCR amplification was found challenging in this study, probably due to the high genomic repetitiveness in the species causing primer mismatches. While these repeats (categorized as transposable elements, tandem repeats, or segmental duplications) serve various evolutionary and regulatory roles, they could explain the off-target retrieval of sequences from different genomic regions, which was a frequent problem encountered in this study. In fact, the particularly large and complex genome of faba bean was estimated to have over 85% of its genome as repetitive elements^20^.

### Conclusion and future prospects

This analysis of the *PEBP* gene family in *Vicia faba* provides valuable insights into the molecular basis of regulation of flowering time and plant architecture in this legume crop which has great potential for increased cultivation globally. The findings lay a strong foundation for future functional studies and targeted breeding efforts aimed at optimizing faba bean varieties for diverse agricultural needs and environmental conditions. Future research should investigate the regulatory networks controlling *VfTFL1* expression, including the role of identified cis-acting elements. It should also explore interactions among *PEBP* family members and their combined effects on plant phenotypes. Additionally, developing more robust molecular markers for determinate growth habit will be valuable, especially given the complex genetic control suggested by our results.

## Electronic supplementary material

Below is the link to the electronic supplementary material.

Supplementary Material 1

Supplementary Material 2

## Data Availability

All data relevant to the reproduction of this study are included in the supplementary material provided or can be requested from authors. The datasets generated and analysed during the current study are available in the BankIt repository under the following accession numbers: PQ878658, PQ878659, PQ878660, PQ878661, PQ878662, PQ878663, PQ878664, PQ878665.
