# Supplementary material for: Comprehensive identification of the PEBP gene family in Vicia faba highlighting VfTFL1a variation and phenotypic insights into determinate growth
Source: Sci Rep. 2025 Aug 6;15:28687. doi: 10.1038/s41598-025-12864-0 (PMC12328713; doi:10.1038/s41598-025-12864-0)
Supplement: Supplementary file 2 — Supplementary Material 2 [file 41598_2025_12864_MOESM2_ESM.pdf]

**Comprehensive Identification of the *PEBP* Gene Family in *Vicia faba* Highlighting *VfTFL1a* Variation and Phenotypic Insights into Determinate Growth**

Hannah Ohm*, Umer Mahmood, Jenny Östberg, Josefin Alverup, Åsa Grimberg, Per Hofvander
Department of Plant Breeding, Swedish University of Agricultural Sciences (SLU), SE-234 22 Lomma, Sweden

* Correspondence:
Hannah Ohm, hannah.ohm@slu.se

| **Supplementary Material/Figure/Table** | **Description** |
|---|---|
| Supplementary Material S1 | *VfTFL1a* Primers |
| Supplementary Material S2 | PEBP Family Information |
| Supplementary Material S3 | PEBP Motif Sequences |
| Supplementary Material S4 | PEBP Alignment |
| Supplementary Table S5 | Predicted Cis-Acting Regulatory Elements |
| Supplementary Table S6 | Coinciding Cis-Acting Regulatory Elements |
| Supplementary Figure S7 | *VfTFL1a* Alignment |
| Supplementary Material S8 | Phenotypic Data |
| Supplementary Figure S9 | Determinate Growth |

Supplementary S4

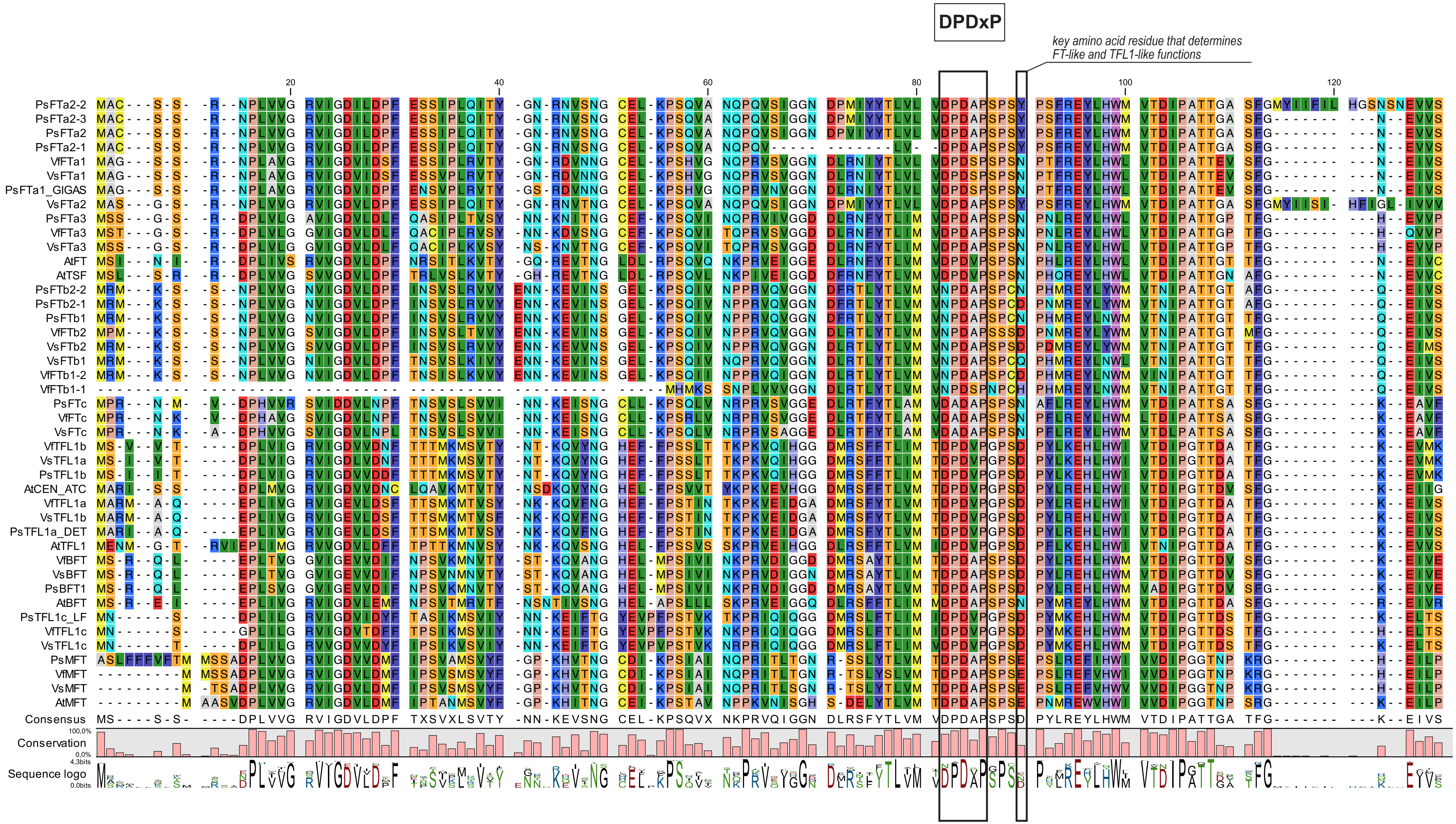

# Supplementary S4

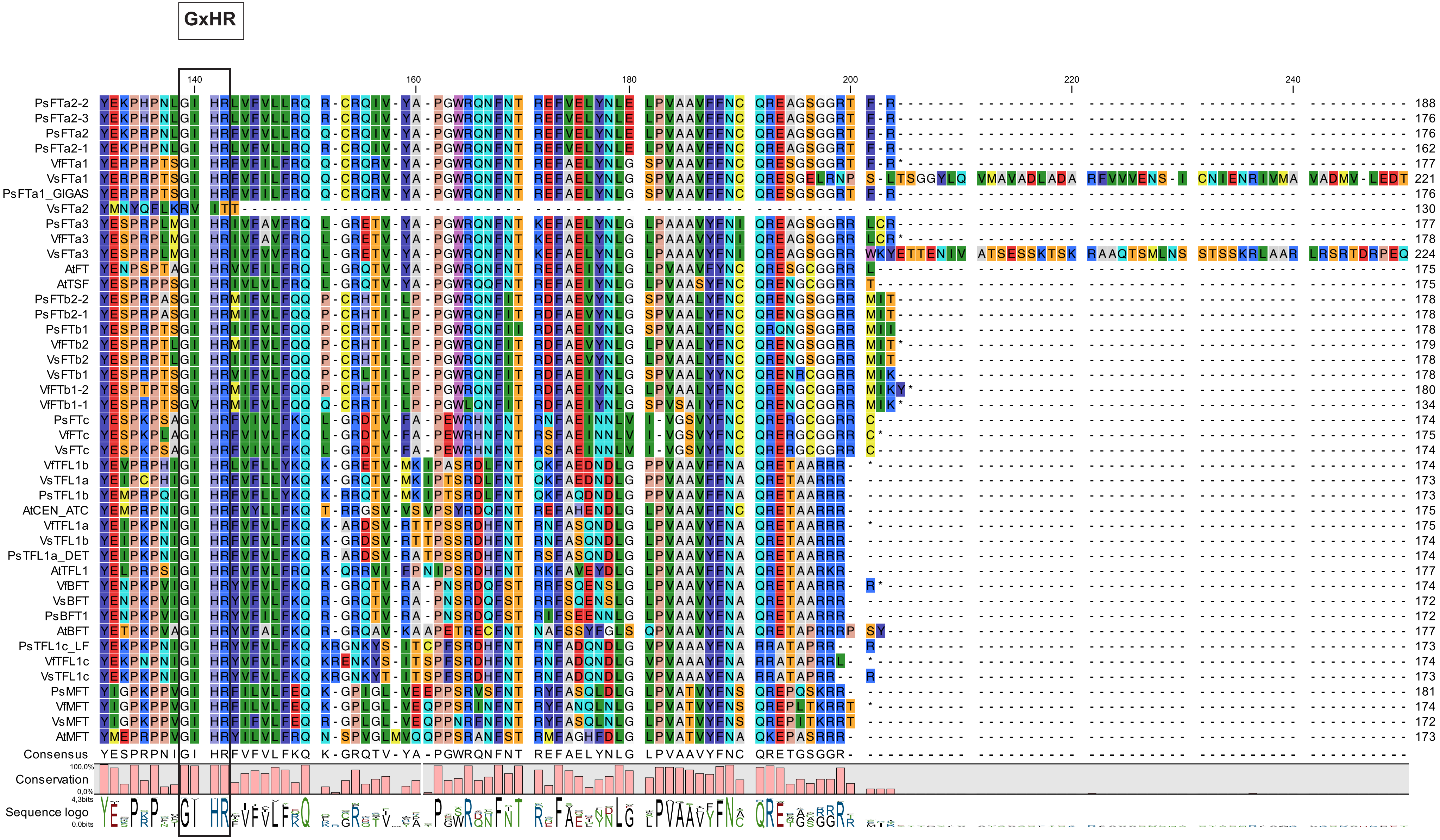

Supplementary S6

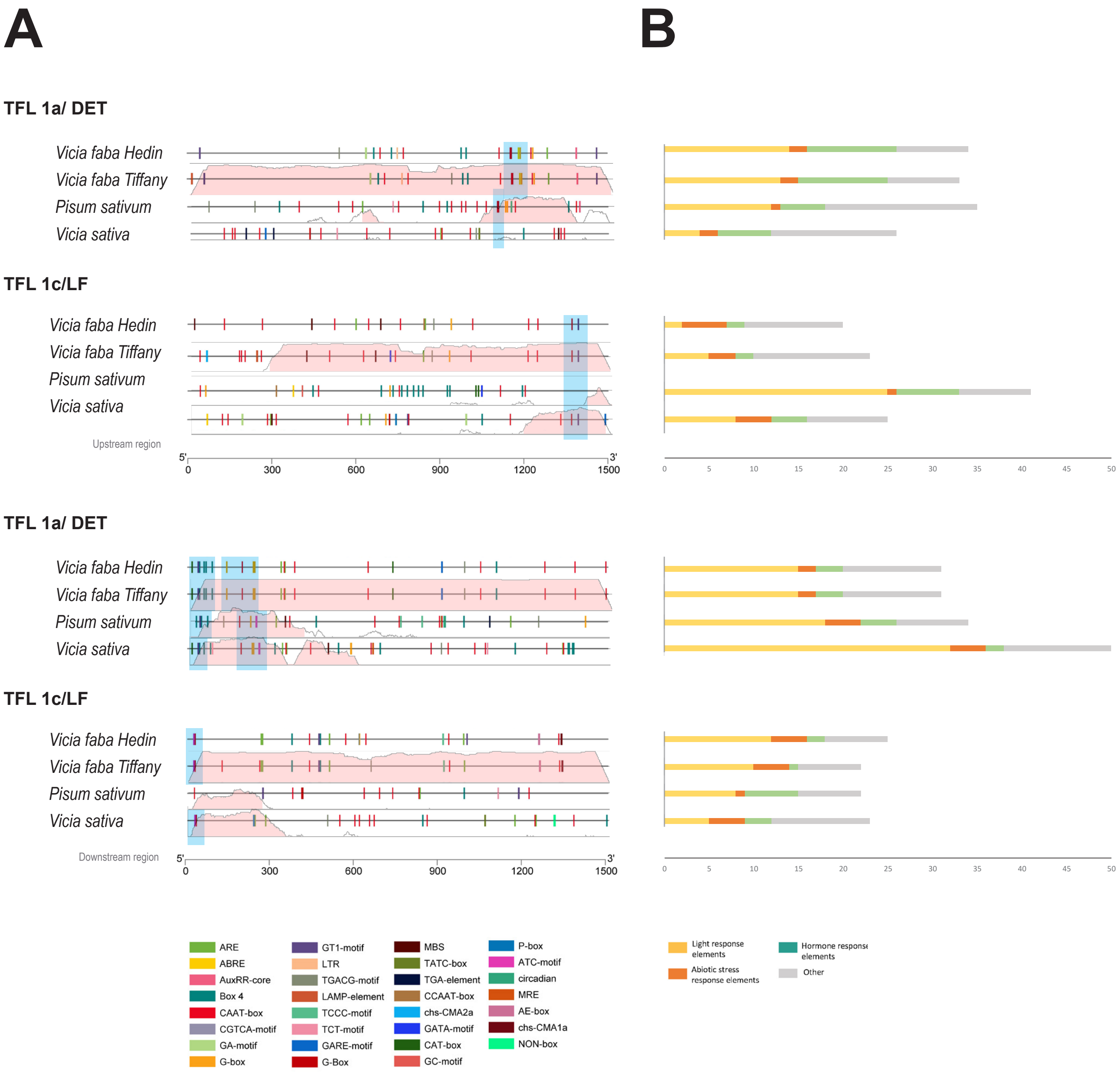

# Supplementary S7

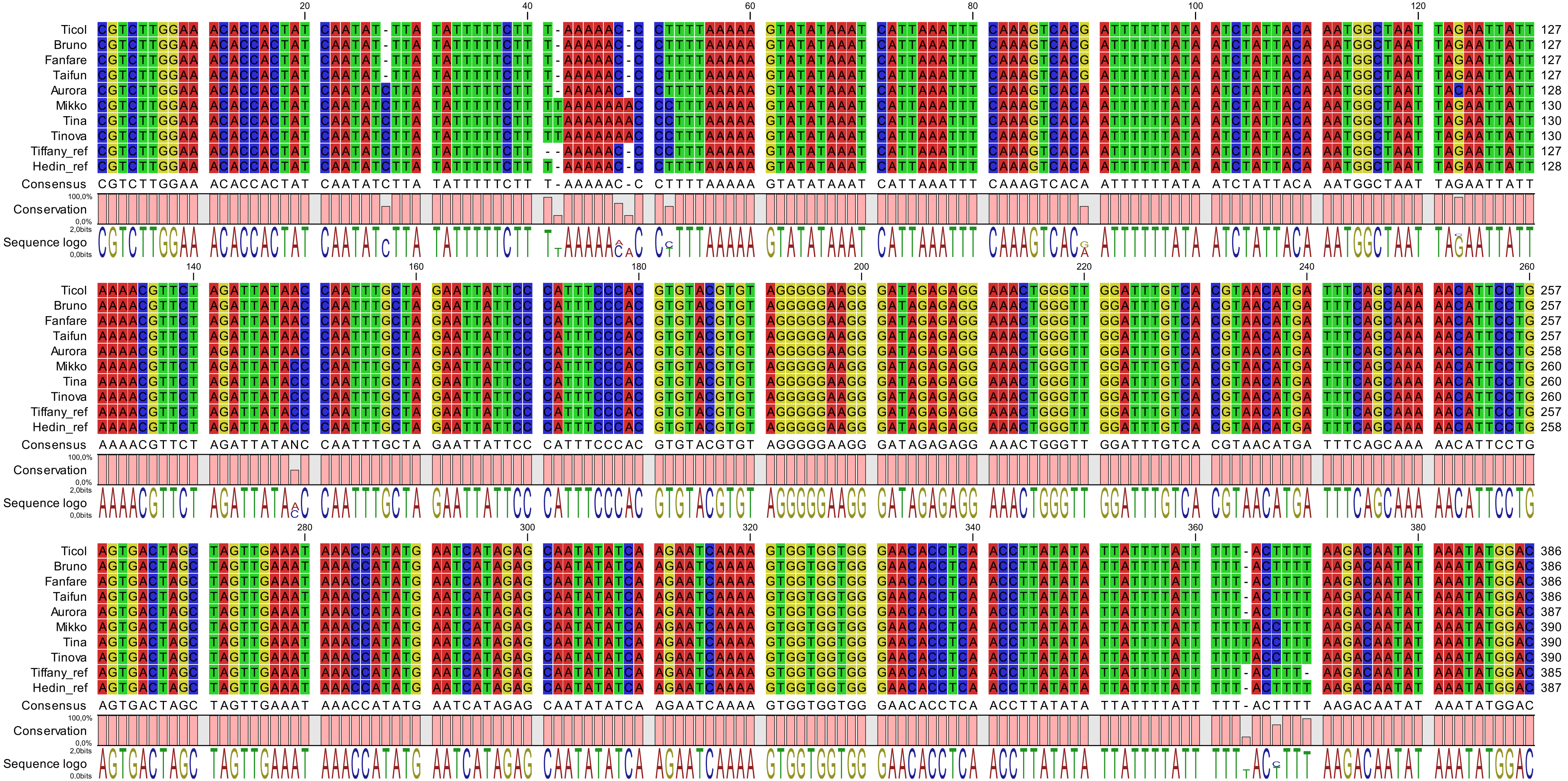

# Supplementary S7

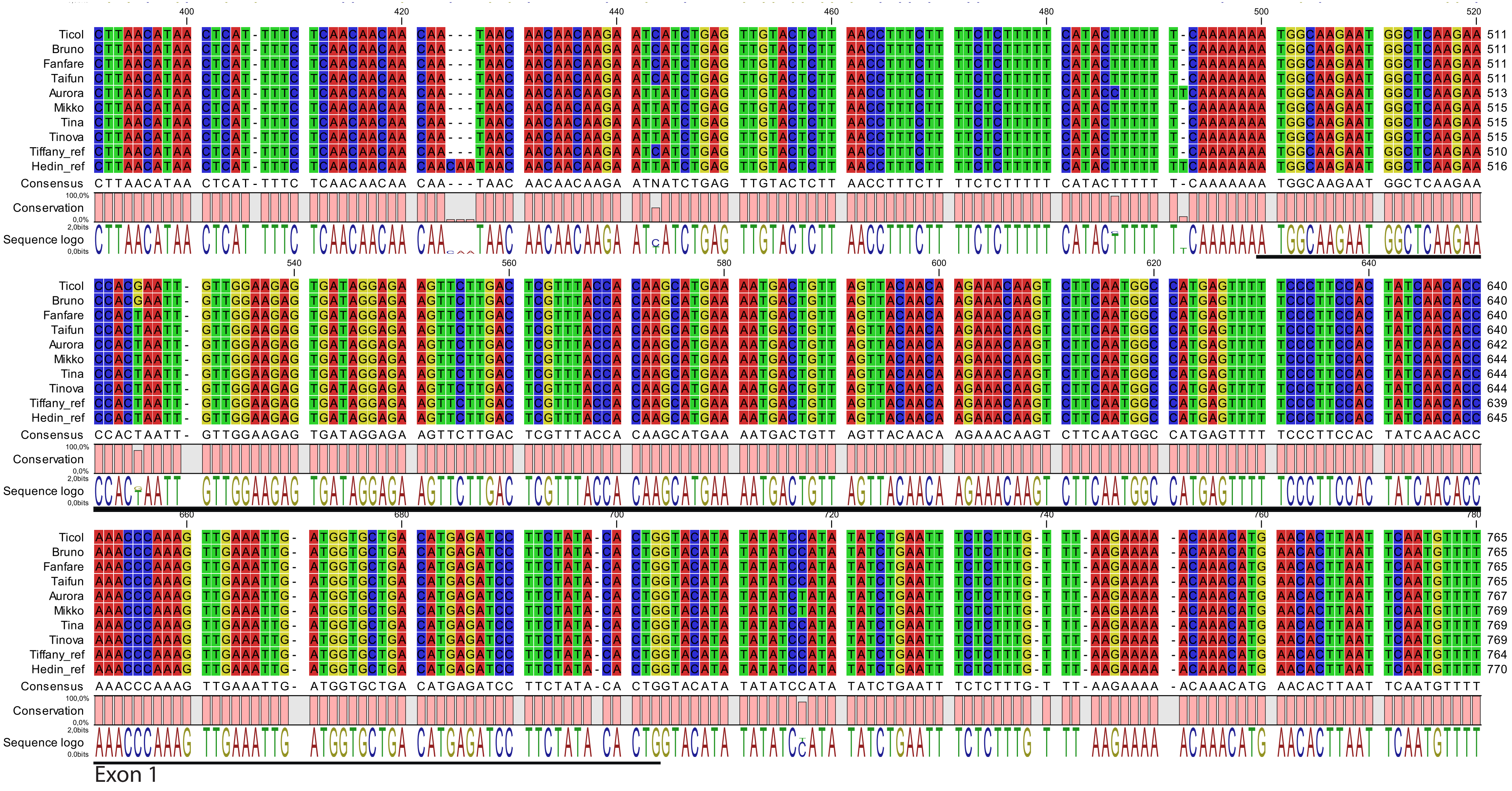

Supplementary S7

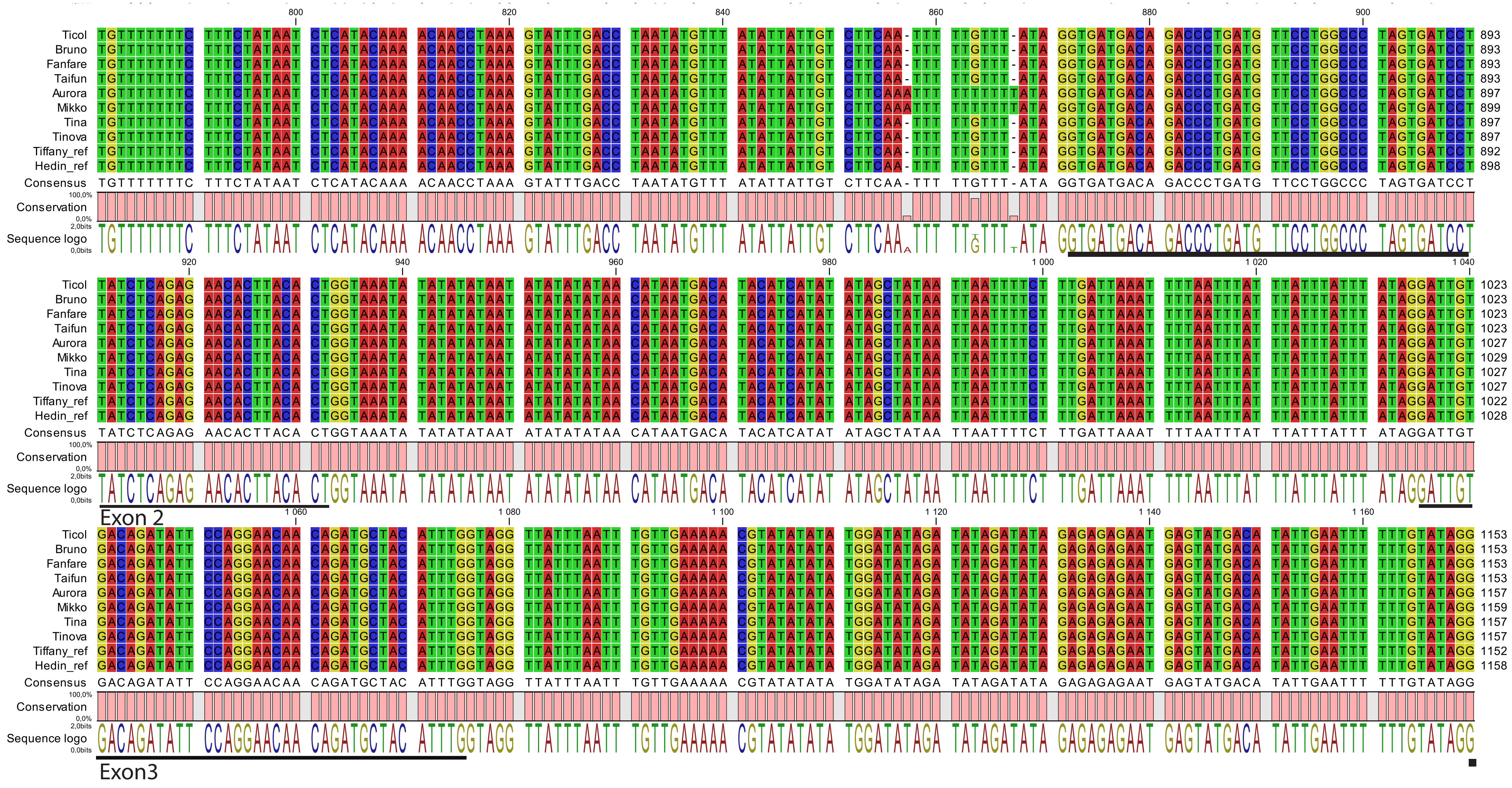

# Supplementary S7

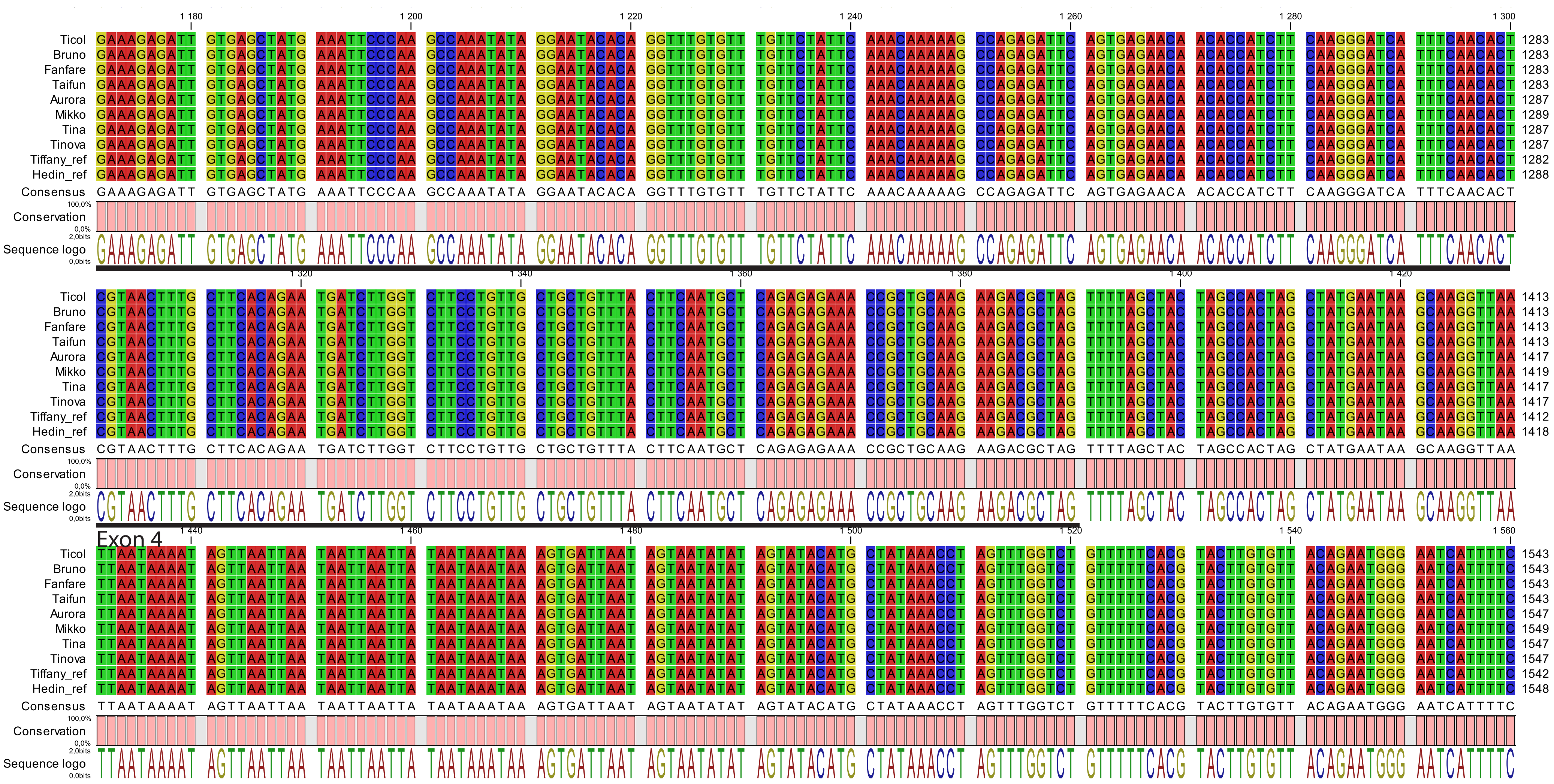

## Supplementary S7

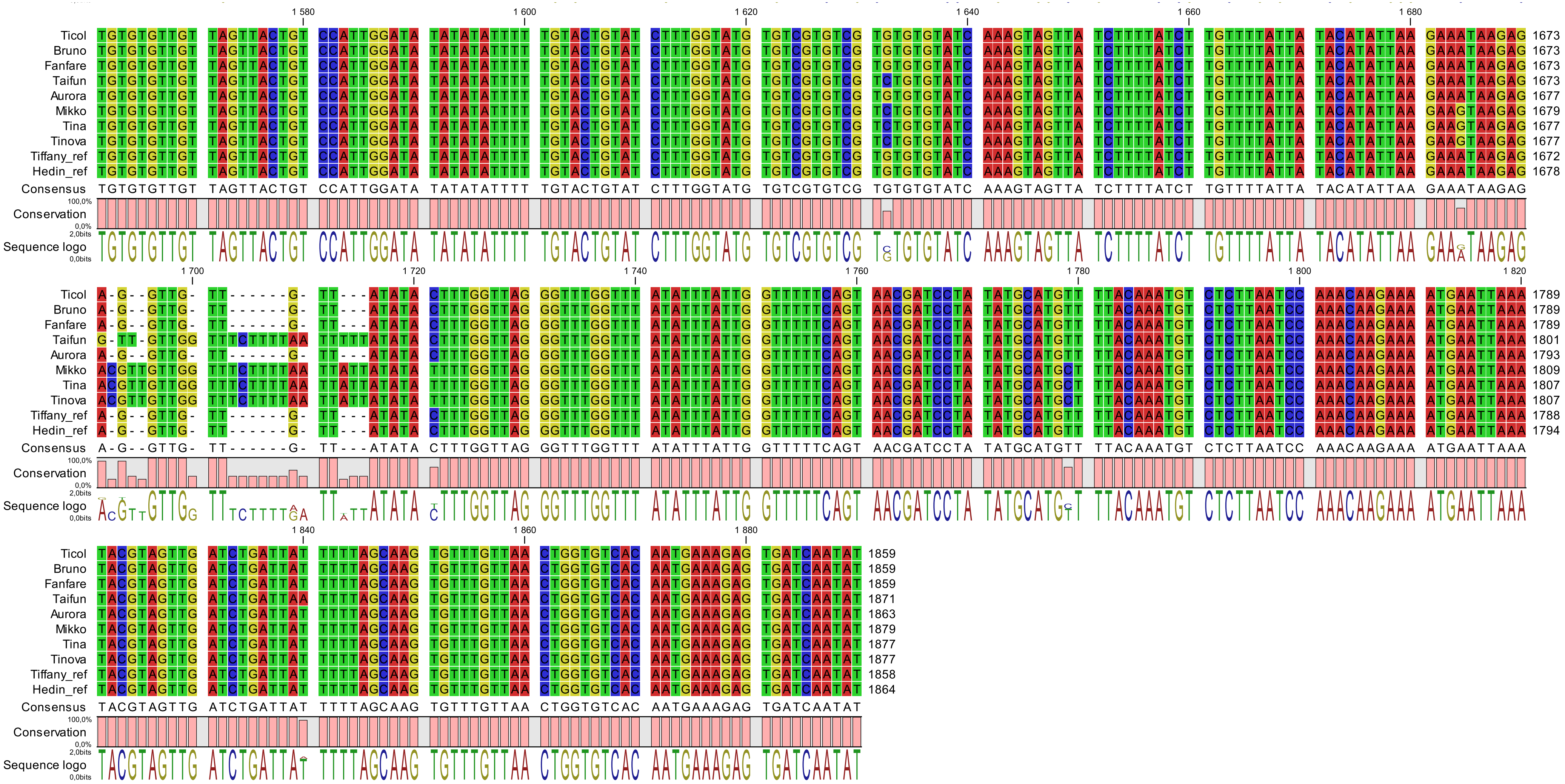

# Supplementary S9

Tinova

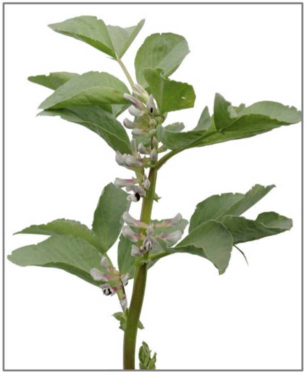

Tina

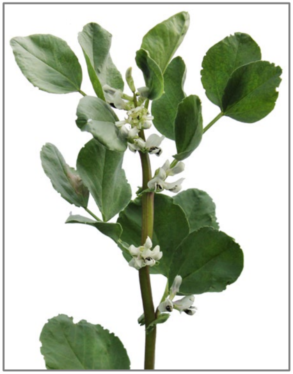

Bruno

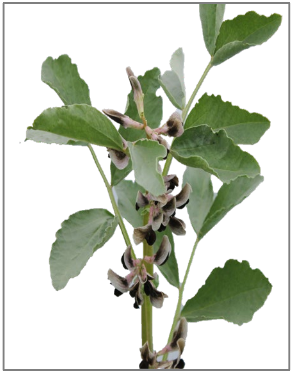

Ticol

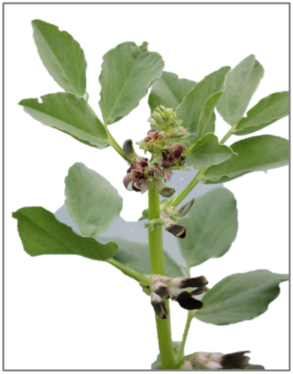

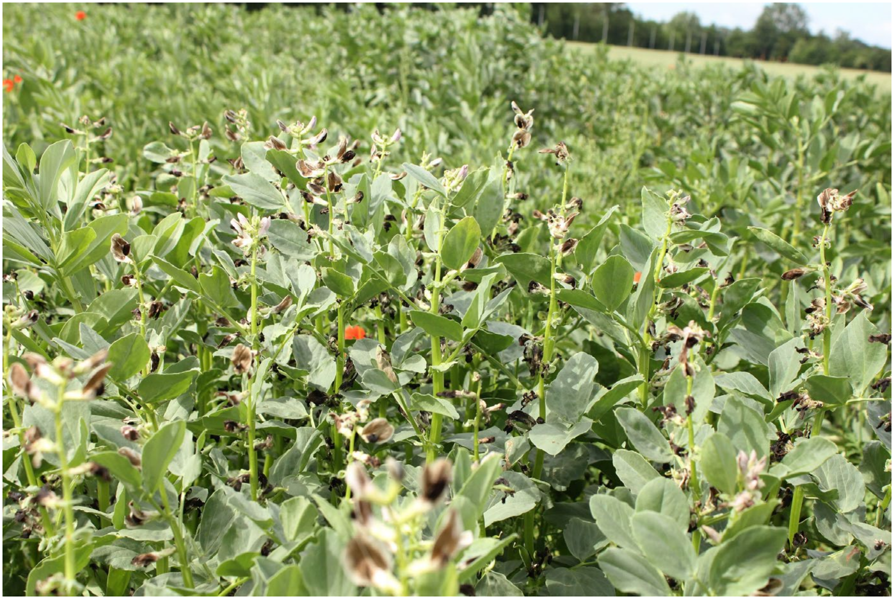

Field plot with Ticol
